# Editorial: Codon Usage and Dinucleotide Composition of Virus Genomes: From the Virus-Host Interaction to the Development of Vaccines

**DOI:** 10.3389/fmicb.2021.791750

**Published:** 2021-11-30

**Authors:** Rosa M. Pintó, Cara C. Burns, Gonzalo Moratorio

**Affiliations:** ^1^Enteric Virus Laboratory, Section of Microbiology, Virology and Biotechnology, Department of Genetics, Microbiology and Statistics, School of Biology, University of Barcelona, Barcelona, Spain; ^2^Division of Viral Disease, Molecular Epidemiology and Surveillance, Centers for Disease Control and Prevention (CDC), Atlanta, GA, United States; ^3^Molecular Virology Laboratory, Experimental Evolution Laboratory, Nuclear Research Centre, School of Sciences, University of la República, Institute Pasteur, Montevideo, Uruguay

**Keywords:** codon usage bias, dinucleotide bias, codon deoptimization, tRNA competition, translation selection, codon usage code for protein folding, mutational robustness

The codon usage and dinucleotide frequencies of an organism depend mostly on its nucleotide composition, but also on the evolutionary forces that have acted on its genome including mutation, genetic drift, and selection. Different selection pressures may contribute to shape the genome composition and codon usage of viruses:

- Codon usage and dinucleotide biases may result from the need to maintain RNA secondary structures involved in splicing and gene expression (Takata et al., 2018).
- Dinucleotide bias may result from the need to evade cell defense mechanisms. UpA, and particularly CpG, dinucleotides may be perceived as pathogen-associated molecular patterns by host cells and consequently their frequencies in viral genomes tend to decrease (Atkinson et al., 2014).
- Codon usage may also result from translation selection. Abundant codons, pairing with abundant tRNAs, would be selected over rare codons, pairing with non-abundant tRNAs, to improve the efficiency and accuracy of translation. On the contrary, rare codons would persist through mutational pressure and genetic drift (Duret, 2002; Hershberg and Petrov, 2008). Viruses do not code for tRNAs, and instead they use the host tRNA pool for their own translation, making even harder to discern the role of selection on shaping their codon usage. Although a similar codon usage between viruses and their hosts may be anticipated, excessive similarity may impede host translation, with the associated deleterious effects; consequently, viruses have evolved to an optimal range of codon usage bias (Moratorio et al., 2013; Chen et al., 2020).
- Codon usage may also be shaped by selection for the control of the translation rate. The scarcity of rare codon pairing tRNAs may result in ribosome stalling, slowing down the mRNA translation speed. In doing so, rare codons may play a role in controlling the co-translational folding (Chaney and Clark, 2015; Yu et al., 2015; Zhao et al., 2017; D'Andrea et al., 2019; Pintó and Bosch, 2021).
- Additionally, codon usage may be selected to maintain mutational robustness. Codons one mutation apart from a stop codon may tend to be avoided in genomes (Moratorio et al., 2017; Carrau et al., 2019).

Noteworthy, based on some of the above principles, candidate poliovirus vaccines have been developed and employed in the final phases of the poliovirus eradication (Burns et al., 2009; Van Damme et al., 2019; Konopka-Anstadt et al., 2020; De Coster et al., 2021; Saez-Llorens et al., 2021; Wahid et al., 2021).

In the present Research Topic, four papers deal with the analysis of the genome composition of viruses in the context of their host interactions, and four additional articles deal with re-coding of virus genomes aiming to synthesize vaccines (Figure 1).

**Figure 1:**
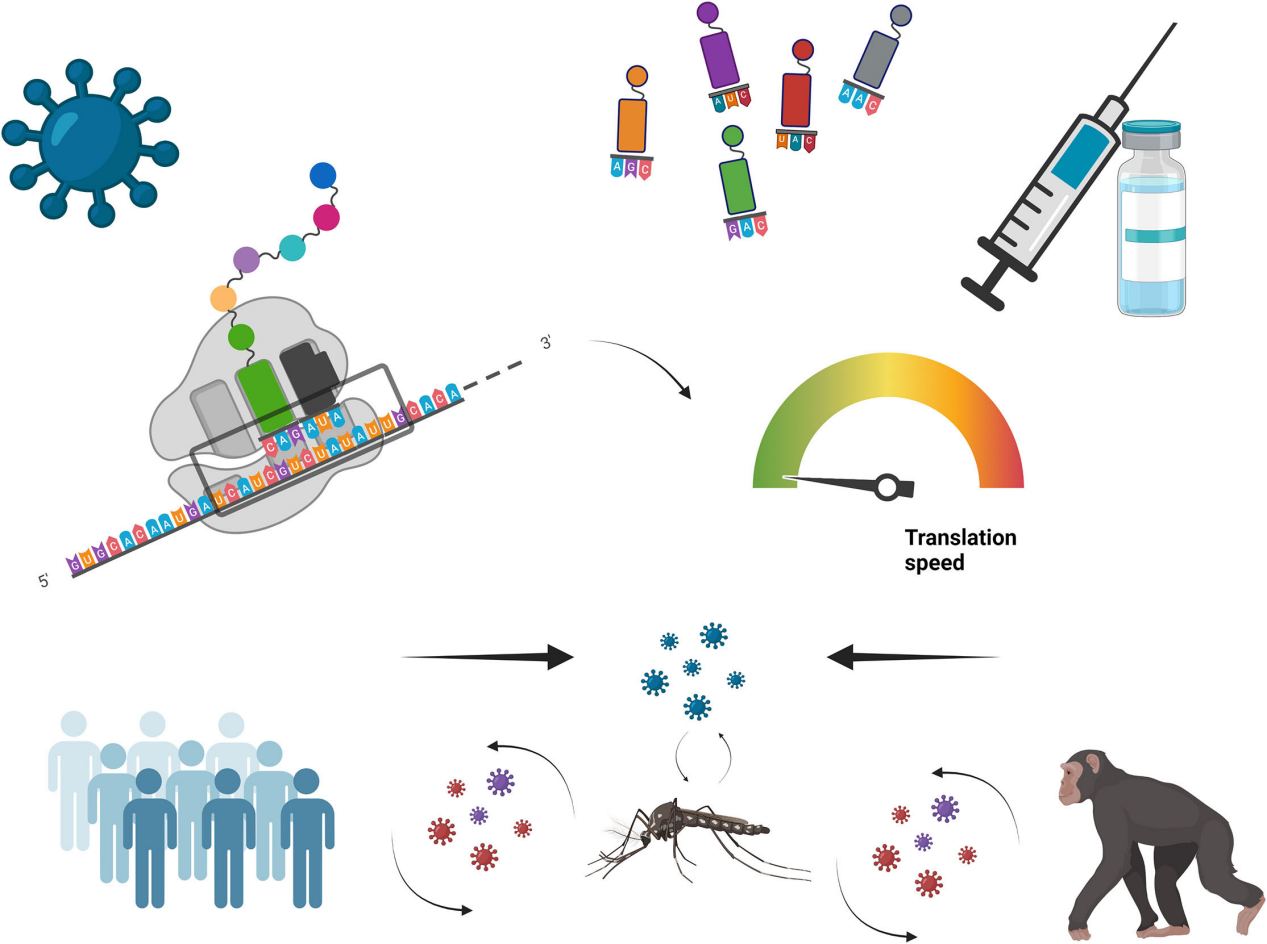
Viral vaccines design based on genome re-coding.

Simón et al. analyze the nucleotide composition and codon usage across 10,000 viruses in comparison with their 1,170 hosts, concluding that overall there is strong similarity in genomic base composition and codon usage between prokaryotes and their phages, but not between eukaryotic viruses and their hosts. However, while this is a general feature of animal and plant viruses, exceptions do exist. For instance, Ata et al., using phylogenetic dependent codon usage analysis, prove the critical role of the virus-host interaction in the evolutionary processes shaping the codon usage of Hantaan virus, which shows antagonistic codon usage preferences between *Homo sapiens* and their natural murine reservoirs (*Apodemus agrarius* and *Niviventer confucianus*). There is generally a fair codon adaptation in humans that is not so evident in rodents. Similarly, Roy et al. compare the codon usage pattern of MERS-CoV, SARS-CoV, and SARS-CoV-2 and found that in this latter case there is a lower degree of adaptation to the human host, suggesting that it could contribute to its lower virulence. In Jordán-Paiz et al., the role of codon usage and CpG dinucleotide content in the HIV-host interaction is reviewed. Codon deoptimization of HIV genes decreases its replication capacity. In HIV-1 the codon-driven attenuation is related with the RNA secondary structure, which regulates splicing and viral gene expression, rather than with the control of the speed of translation through tRNAs availability. In fact, late in infection HIV-1 alters the cellular tRNA pool enriching it in rare human tRNAs to match the codon usage of the late genes. On the other hand, increases of the CpG content reduce the HIV-1 replication. One explanation is related with the binding of CpG-rich mRNAs with the innate response factor ZAP; another reason could be the changes in RNA splicing and RNA secondary structure induced by the higher CpG content.

In the second part of the Topic, Pereira-Gómez et al. elegantly review genome recoding strategies for viral attenuation from an evolutionary perspective. These strategies include alteration of dinucleotide frequencies, deoptimization of codons and codon pairs, and decrease of viral mutational robustness. A very nice example of codon deoptimization is the development of live attenuated strains of foot and mouth disease virus (FMDV) described in Diaz-San Segundo et al. The strategy was based on the deoptimization of the non-structural proteins coding regions, which are more conserved than the capsid coding region, to provide a backbone for the development of multiple-serotype live attenuated vaccines. Giménez-Roig et al. review the approaches employed for the development of adenoviral-based vaccine, and they discuss a provocative novel strategy using codon optimization of the transgene, pursuing a good balance between transgene expression and virus attenuation through the competition for tRNAs. Finally, Chavarria-Miró et al. describe the growth characteristics of a hepatitis A virus strain isolated through genomic selection, which contains epistatic mutations located at the Internal Ribosome Entry Site (IRES) and in the capsid coding region, resulting in a fast growing virus. While IRES mutations lead to a more active initiation of translation, the right combination of deoptimized and optimized codons in the capsid coding region results in a correct capsid folding despite a faster translation. Overall, this strain shows a higher virus production in a shorter time compared to the standard virus strain presently used in vaccine production from which it derived.

The findings and conclusions in this report are those of the authors and do not necessarily represent the views of the Centers for Disease Control and Prevention.

## Author Contributions

RP wrote the Editorial. CB and GM contributed to the final version of the Editorial. All authors contributed to the article and approved the submitted version.

## Conflict of Interest

The authors declare that the research was conducted in the absence of any commercial or financial relationships that could be construed as a potential conflict of interest.

## Publisher's Note

All claims expressed in this article are solely those of the authors and do not necessarily represent those of their affiliated organizations, or those of the publisher, the editors and the reviewers. Any product that may be evaluated in this article, or claim that may be made by its manufacturer, is not guaranteed or endorsed by the publisher.
